# *In Vitro* Evaluation of Biocompatibility of Uncoated Thermally Reduced Graphene and Carbon Nanotube-Loaded PVDF Membranes with Adult Neural Stem Cell-Derived Neurons and Glia

**DOI:** 10.3389/fbioe.2016.00094

**Published:** 2016-12-06

**Authors:** Çağla Defteralı, Raquel Verdejo, Shahid Majeed, Adriana Boschetti-de-Fierro, Héctor R. Méndez-Gómez, Eva Díaz-Guerra, Daniel Fierro, Kristian Buhr, Clarissa Abetz, Ricardo Martínez-Murillo, Daniela Vuluga, Michaël Alexandre, Jean-Michel Thomassin, Christophe Detrembleur, Christine Jérôme, Volker Abetz, Miguel Ángel López-Manchado, Carlos Vicario-Abejón

**Affiliations:** ^1^Instituto Cajal, Consejo Superior de Investigaciones Científicas (IC-CSIC), Madrid, Spain; ^2^Centro de Investigación Biomédica en Red sobre Enfermedades Neurodegenerativas (CIBERNED-ISCIII), Madrid, Spain; ^3^Instituto de Ciencia y Tecnología de Polímeros (ICTP-CSIC), Madrid, Spain; ^4^Helmholtz-Zentrum Geesthacht (HZG), Zentrum für Material- und Küstenforschung GmbH, Institut für Polymerforschung, Geesthacht, Germany; ^5^Department of Chemistry, Center for Education and Research on Macromolecules (CERM), University of Liège, Liège, Belgium

**Keywords:** uncoated graphene, PVDF membranes, carbon nanotubes, adult neural stem cells, neurons, synapses, oligodendrocytes

## Abstract

Graphene, graphene-based nanomaterials (GBNs), and carbon nanotubes (CNTs) are being investigated as potential substrates for the growth of neural cells. However, in most *in vitro* studies, the cells were seeded on these materials coated with various proteins implying that the observed effects on the cells could not solely be attributed to the GBN and CNT properties. Here, we studied the biocompatibility of uncoated thermally reduced graphene (TRG) and poly(vinylidene fluoride) (PVDF) membranes loaded with multi-walled CNTs (MWCNTs) using neural stem cells isolated from the adult mouse olfactory bulb (termed aOBSCs). When aOBSCs were induced to differentiate on coverslips treated with TRG or control materials (polyethyleneimine-PEI and polyornithine plus fibronectin-PLO/F) in a serum-free medium, neurons, astrocytes, and oligodendrocytes were generated in all conditions, indicating that TRG permits the multi-lineage differentiation of aOBSCs. However, the total number of cells was reduced on both PEI and TRG. In a serum-containing medium, aOBSC-derived neurons and oligodendrocytes grown on TRG were more numerous than in controls; the neurons developed synaptic boutons and oligodendrocytes were more branched. In contrast, neurons growing on PVDF membranes had reduced neurite branching, and on MWCNTs-loaded membranes oligodendrocytes were lower in numbers than in controls. Overall, these findings indicate that uncoated TRG may be biocompatible with the generation, differentiation, and maturation of aOBSC-derived neurons and glial cells, implying a potential use for TRG to study functional neuronal networks.

## Introduction

Graphene, graphene-based nanomaterials (GBNs), and carbon nanotubes (CNTs) are being investigated for their potential applications in tissue engineering and regenerative medicine (Novoselov et al., 2004; John et al., 2015; Marchesan et al., 2015, 2016; Defterali et al., 2016; Lopez-Dolado et al., 2016; Zhou et al., 2016). In fact, scaffolds that support neural growth can be made of porous foams and membranes alone or loaded with a variety of GBNs and CNTs (Ramanathan et al., 2008; Chao et al., 2010; Alvarez et al., 2013; Li et al., 2013; Shah et al., 2014; Song et al., 2014; Weaver and Cui, 2015; Akhavan et al., 2016; Guo et al., 2016b,c). Moreover, there is evidence indicating that graphene and GBNs can serve as substrates for neural stem cells (NSC) differentiation, neuronal and oligodendrocyte growth, and the formation of neural circuits in cell culture (*in vitro)* (Li et al., 2011, 2013; Park et al., 2011; Akhavan and Ghaderi, 2013a,b, 2014; Lorenzoni et al., 2013; Solanki et al., 2013; Tang et al., 2013; Akhavan et al., 2014, 2015; Shah et al., 2014). In these previous studies, cells were either seeded on graphene or on GBNs coated with proteins such as laminin and synthetic polymers such as poly-lysine, substances which are known to promote cell adhesion and neurite outgrowth (Vicario et al., 1993; Calof et al., 1994; Otaegi et al., 2007; Nishimune et al., 2008). In addition, cells were plated on graphene composites, graphene oxides, or on reduced graphene oxides with different surface charges and degree of electrical, photo, and laser stimulation (Akhavan and Ghaderi, 2013a,b, 2014; Tu et al., 2013a, 2014; Akhavan et al., 2014, 2015; Guo et al., 2016a). Similarly, both uncoated and coated functionalized single-walled CNTs (SWCNTs) and multi-walled CNTs (MWCNTs) as well as aligned CNTs and nanofibers have been reported to permit and stimulate neuronal growth and the formation of active synaptic contacts (Jan and Kotov, 2007; Malarkey et al., 2009; Cellot et al., 2011; Jin et al., 2011; Fabbro et al., 2013; Gupta et al., 2015; Vicentini et al., 2015).

In spite of these potential applications, other studies have reported that GBNs can cause cytotoxic and genotoxic effects on cell lines (PC12, neuroblastoma, and A549 cells), mesenchymal stem cells (Zhang et al., 2010; Chang et al., 2011b; Akhavan et al., 2012; Lv et al., 2012; Bianco, 2013; Tu et al., 2013b), and neurons (Bramini et al., 2016). CNTs, particularly if used as produced materials, can also induce toxic effects on neural cells in part due to the presence of CNT aggregates, impurities such as amorphous carbon and metallic nanoparticles (Jakubek et al., 2009; Cellot et al., 2010; Wu et al., 2012; Chen et al., 2013; Meng et al., 2013; Bussy et al., 2015). However, recent studies indicate that chemical functionalization can reduce toxicity while preserving the highly conductive character of CNTs (John et al., 2015; Oliveira et al., 2015; Marchesan et al., 2016).

To the best of our knowledge, no studies reporting the biocompatibility of uncoated graphene with adult NSCs (aNSCs) *in vitro* have yet been published. Moreover, very few works have addressed the effect of uncoated graphene on the growth of neurons and glial cells. They reported that neurons can develop on graphene but their attachment was reduced compared to when the neurons were grown on poly-d-Lysine and laminin (Bendali et al., 2013; Sahni et al., 2013), that graphene stimulated neurite length compared to a glass substrate (Lee et al., 2015), or that pristine graphene and graphene-based substrates were permissive for neuronal outgrowth (Veliev et al., 2016) and synapse formation and function (Fabbro et al., 2016).

In the present study, we have investigated the effects of uncoated thermally reduced graphene (TRG) (Defterali et al., 2016) on the proliferation and differentiation potential of cultured adult mouse olfactory bulbs (aOBSCs), a population of previously characterized aNSCs (Vergaño-Vera et al., 2009; Nieto-Estévez et al., 2013) as well as on neuronal and glial survival and maturation. Since membranes are being used to make biocompatible neural scaffolds (see above), the differentiation of aOBSCs was also tested on pristine poly(vinylidene fluoride) (PVDF) membranes and on PVDF membranes loaded with MWCNTs. Our findings indicate that uncoated TRG is a permissive material that allows for the multi-lineage differentiation of cultured aOBSCs into neurons, astrocytes, and oligodendrocytes and the synaptic maturation of aOBSC-derived neurons. They also show that TRG supports the morphological differentiation of aOBSC-derived oligodendrocytes. In contrast, the morphological differentiation of aOBSC-derived neurons and oligodendrocyte survival were reduced when seeded on the PVDF membranes.

## Materials and Methods

### Animals

All animal care and handling was carried out in accordance with European Union guidelines (directive 2010/63/EU) and Spanish legislation (Law 32/2007 and RD 53/2013), and the protocols were approved by the Ethical Committee of the Consejo Superior de Investigaciones Científicas (CSIC) and Comunidad de Madrid. Food and water were administered *ad libitum* and environmental conditions were strictly controlled: 12-h light/dark cycle, temperature 22°C, and humidity 44%. All efforts were made to ameliorate the suffering of the animals.

### Preparation of TRG and Coating of Glass Coverslips

**Figure 1:**
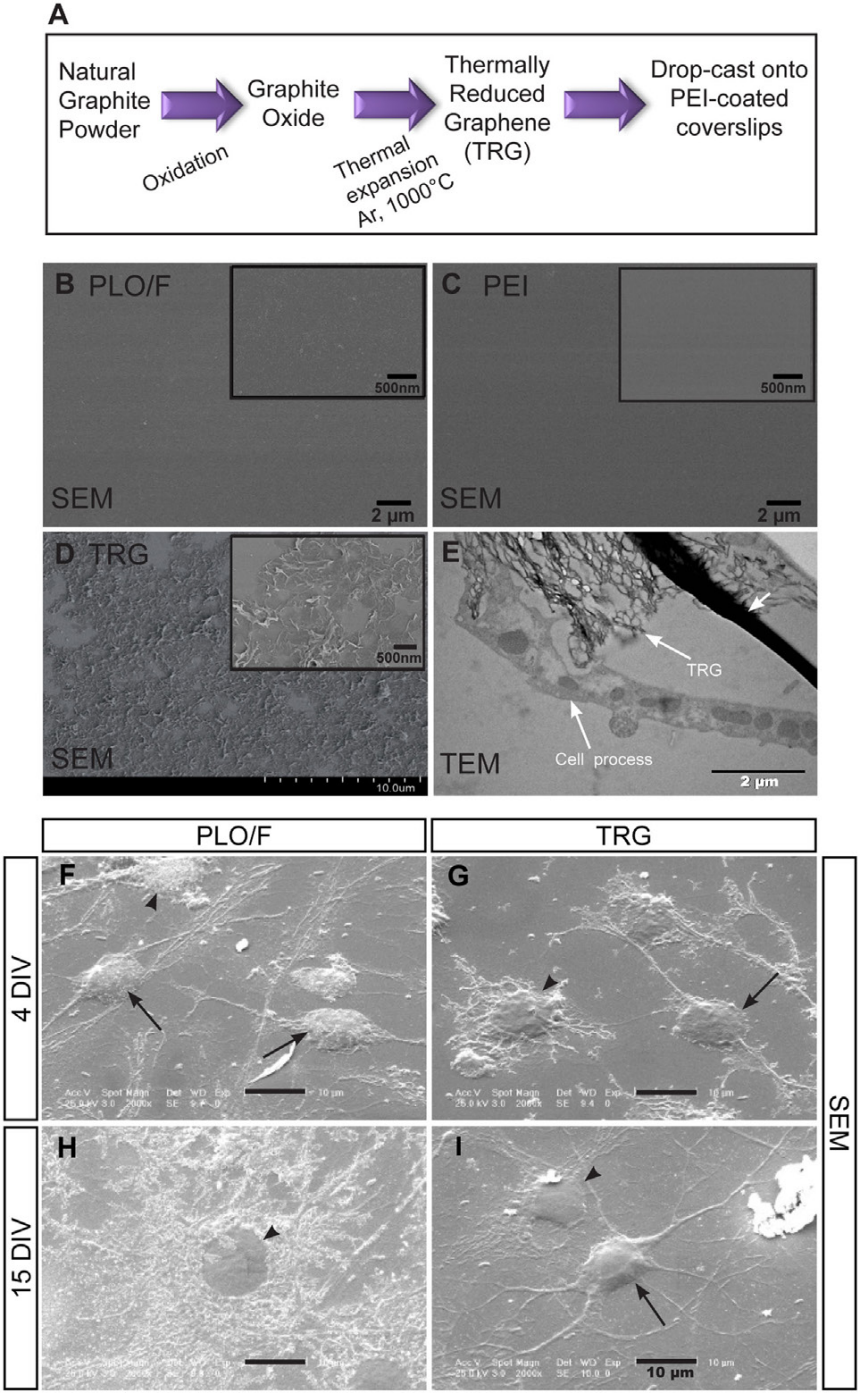
**The distribution of PLO/F, PEI, and TRG on glass coverslips and the growth of aOBSC-derived neurons and glia on these materials**. The scheme summarizes the preparation of TRG and coating of glass coverslips **(A)**. SEM microphotographs of coverslips coated with PLO/F and PEI are shown prior to seeding **(B,C)**. SEM microphotograph of the TRG coating reveals the wrinkle structures of TRG and that the material is relatively uniformly distributed and is almost completely covering the coverslip, prior to cell seeding **(D)**. The insets in **(B–D)** show high magnification images. The TEM photograph **(E)** shows the close contact between the cell processes and the TRG (indicated by arrows). The small arrow points to artifacts due to the technique used. The SEM microphotographs show various healthy, differentiated cells on PLO/F-coated coverslips **(F,H)** and TRG-covered coverslips **(G,I)** after 4 DIV and 15 DIV. For images **(F–I)**, arrows indicate cells having neuronal morphology whereas the arrowheads point to glial morphologies.

Graphite oxide (GO) was produced from natural graphite powder (universal grade, 200 mesh, 99.9995%) according to the Brödie method (Schniepp et al., 2006; Verdejo et al., 2008; Romasanta et al., 2011; Defterali et al., 2016). TRG was then formed by the rapid thermal expansion of GO at 1,000°C under argon atmosphere (Figure 1A). This reduction process is not complete and some remaining epoxy, hydroxyl, and carboxyl groups are present on the surface of graphene, resulting in TRG, also called functionalized graphene sheets (Schniepp et al., 2006; Ramanathan et al., 2008; Rao et al., 2009; Zhao et al., 2012; Botas et al., 2013). The nature and the relative amount of oxygen-containing functional groups present on the TRG were analyzed by X-ray photoelectron spectroscopy (XPS). The XPS spectra were recorded using an Escalab 200R spectrometer with a hemispherical analyzer operated on a constant pass energy mode and non-monochromatized Mg KR X-ray radiation (hν = 1253.6 eV) at 10 mA and 12 kV. Data analysis was performed with the “XPS peak” program. The spectra were decomposed by the least-squares fitting routine using a Gauss/Lorentz product information after subtracting a Shirley background.

Raman measurements were performed on a Renishaw Invia Raman Microscope. The analyses were done using an argon laser at 514.5 nm excitation wavelength and 0.02 cm^−1^ resolution. TRG powder was placed on a glass slide and air-dried before the measurements were taken. The morphology of the TRG was analyzed by transmission electron microscopy (TEM) on a Philips Tecnai 20 TEM apparatus using a voltage of 200 kV. The samples were prepared by immersion of the TEM grid in a dilute solution of nanoparticles in tetrahydrofuran (THF) and letting the solvent evaporate.

This material was dispersed for 5 min in dimethylformamide (DMF, 4 mg/ml) using an ultrasonication probe (Sonics Vibracell VC505), and the TRG/DMF solution was then centrifuged at 3,000 rpm for 1 h. Round glass coverslips (12 mm diameter) were coated with 0.5 wt% PEI electrolyte in water by immersion for 2 h at 60°C. Excess PEI solution was removed and 25 μL of a TRG/DMF solution was drop-cast onto the dried PEI-coated coverslips (Figure 1A). Finally, the DMF was evaporated and the coverslips were placed in an air circulating oven to ensure complete removal of the DMF solvent. The previous coating of the coverslips with PEI was necessary to attach the TRG to the glass coverslip. The TRG was analyzed before and after seeding and fixing the cells by TEM and scanning electron microscopy (SEM; Hitachi, SU8000).

### Preparation and Characterization of PVDF Membranes and PVDF Membranes Loaded with CNTs

Poly(vinylidene fluoride) Kynar^®^ F6000, with an average molecular weight of 250 kg/mol was obtained from Atofina Chemicals Inc., and Lithium chloride (LiCl), *N*,*N*-dimethylacetamide (DMAc) were purchased from Merck. The MWCNTs obtained by chemical vapor deposition with purity of >98%, surface area of 250 m^2^/g, tube diameter in the range of 12–15 nm and 8–12 walls were provided by FutureCarbon GmbH.

#### Functionalization of MWCNTs

The poly(methyl methacrylate) (PMMA) chains for MWCNTs functionalization were synthesized by nitroxide-mediated polymerization (NMP) and end-capped by a cleavable alkoxyamine (PMMA-ONR_2_). PMMA grafting onto MWCNTs was achieved following this protocol: 0.075 g (0.426 mmol) of 2-methyl-3-nitro-2-nitrosopropionate and 0.13 g (0.421 mmol) of 2,2′-azobis(4-methoxy-2,4-dimethylvaleronitrile) were taken in a 500 ml round bottomed flask and degassed by several repeating vacuum-nitrogen cycles. Degassed methyl metacrylate (200 ml, 1.88 mol) was added; the reaction mixture was heated at 50°C for 3 h and then diluted with THF. The polymer was precipitated in methanol, filtered, and dried under vacuum. One gram of MWCNTs and 10 g of PMMA-ONR_2_ were taken in a 250-ml round bottomed flask, and this mixture was degassed by several vacuum-nitrogen cycles. After adding 100 ml of degassed toluene, the mixture was sonicated for 5 min and then heated under vigorous stirring (1000 rpm) at 50°C for 17 h. Functionalized MWCNTs were washed from non-grafted polymer with toluene and vacuum dried at 80°C for 24 h. The number average molecular weight of PMMA-ONR_2_ was 26 kg/mol and a polydispersity of 1.3. The functionalized PMMA-gt-MWCNTs were characterized by thermogravimetric analysis (TGA; Universal V4.7A Instruments).

#### Membrane Preparation

The PVDF membranes were prepared at pilot scale. The cast solution for pristine PVDF membranes contained 5.6 wt% LiCl, 78.4 wt% dimethylacetamide (DMAc), and 16 wt% PVDF. MWCNTs-blended membranes were obtained by loading MWCNTs with respect to PVDF. In order to prepare the solution, LiCl was dissolved in DMAc followed by the addition of MWCNTs. Tip sonication was applied for 45 min to disperse MWCNTs in the mixture. Later, the mixture was subjected to bath sonication for 3 h simultaneously under mechanical stirring (500 rpm) at 60°C. Finally, PVDF was added and the mixture was stirred for 24 h at 60°C. After cooling to room temperature, the solution was evacuated to remove any bubbles. Solution was cast on non-woven polyester support. The cast solution was coagulated at 20°C and nascent membrane was washed at 60°C and dried at 100°C.

#### Membrane Characterization

##### Scanning Electron Microscopy

Scanning electron microscope studies on the membranes were carried out with a LEO Gemini 1550 VP (Zeiss) with field emission cathode operated at 1–1.5 kV. The membrane sample preparation for cross-section analysis was done under cryogenic conditions and samples were sputtered by a very thin layer of Au/Pd.

##### Optical Microscopy

Optical microscopy images were taken with a Leica DMLM, and samples were imaged under reflection mode.

##### Bubble Point Measurements

The surface pore size was analyzed by pore meter 4.900 (PorousMaterial). The membrane stamps of a diameter of 2.5 cm were dipped in a liquid (Porewick^®^) of known surface tension (16 dyn/cm). N_2_ was kept flowing with gradually increased pressure. The driving force (pressure) takes out the liquid from membrane pores. The pressure at which the liquid comes out of the first membrane pore is known as the bubble point, and it was used to calculate the pore size.

##### Water Permeance Measurements

The membranes were analyzed for water permeance at a temperature of 22°C and transmembrane pressure of 2 bar. The water permeance was calculated by the equation: *L* = *V*/(*A*⋅*t*⋅ΔP), where *L* is permeance, *V* is the volume of the permeate, *A* is the membrane area, *t* is time during which the specific permeate volume was measured, and δP is the transmembrane pressure. Permeance was measured as L/(m^2^ h bar).

Once the membranes were obtained, they were cut into circles using a punch and fixed on top of glass coverslips using paraffin wax, which had been sterilized before using UV light.

### Neural Stem Cell Cultures

Adult mouse olfactory bulbs were prepared from 6- to 15-month-old CD1 mice following procedures that were in accordance with national and European Union guidelines for animal care and handling. The cells were plated and expanded as neurospheres in uncoated plastic dishes, a condition that supports the optimal growth of aOBSCs (Vergaño-Vera et al., 2009), and aOBSCs passaged 3–12 times were used in the experiments described here.

To test the effect of TRG on neurosphere formation and growth, cell suspensions were seeded at a density of 5,000 cells/cm^2^ on TRG-coated coverslips and on coverslips coated with 15 μg/ml PLO and 1 μg/ml F (PLO/F), placed in 24-well culture plates (Vicario-Abejón et al., 2003; Vergaño-Vera et al., 2009). Newly formed neurospheres were incubated for 20 h with 5 μM 5′-bromo-2-deoxyuridine (BrdU; Roche) to measure proliferation. Subsequently, neurospheres were collected on matrigel (BD Biosciences) for 30 min, fixed with 4% paraformaldehyde (PFA) for 30 min, and immunostained.

For cell differentiation assays (Table 1), the neurospheres maintained on uncoated plastic dishes (Vergaño-Vera et al., 2009; Nieto-Estévez et al., 2013) were mechanically disaggregated and plated at a density of 100,000–125,000 cells/cm^2^ on glass coverslips coated with PLO/F (our standard coating) PEI or TRG. As mentioned before, the TRG was attached to glass coverslips that were previously coated with PEI and as such the coverslips coated only with PEI were the proper control condition. Moreover, we decided to use another control, i.e., PLO/F-coated coverslips since this is the standard coating in our assays for aOBSC differentiation. Thus, the effects of TRG were compared to both PEI-coated and PLO/F-coated coverslips. The cells were maintained for 3–4 days (short-term differentiation or STD) in serum-free Dulbecco’s modified Eagle medium (DMEM)/nutrient mixture F12 (F12), supplemented with N2 and B27 (DMEM/F12/N2/B27), after which they were fixed with 4% PFA and immunostained. Alternatively, dissociated cells were plated and maintained for 7 days (medium-term differentiation, MTD), or 15–18 days (long-term differentiation, LTD) (Table 1) in DMEM/F12/N2/B27, with the addition of 2% fetal bovine serum (FBS) and when necessary, 20 ng/ml of brain-derived neurotrophic factor (BDNF, Peprotech) to enhance cell survival and maturation (Vergaño-Vera et al., 2009).

**Table 1:** **Protocols used to differentiate aOBSCs and the assays performed**.

Differentiation condition	DIV	Assays	2% FBS
Short term (STD)	3–4	Multi-lineage differentiation	No
		Cell interphase SEM	
Medium term (MTD)	7	Gene expression	Yes
		Cell interphase TEM	
		Cell survival and morphology	
Long term (LTD)	15–18	Cell survival, morphology and synaptic maturation	Yes
		Cell interphase SEM	

*DIV, days in vitro; FBS, fetal bovine serum; SEM, scanning electron microscopy; TEM, transmission electron microscopy*.

For cell differentiation assays in the PVDF membranes, neurospheres were split and dissociated cells seeded at a density of 100,000–125,000 cells/cm^2^ in DMEM/F12/N2/B27/2% FBS for 7 days, after which they were fixed and immunostained.

### Immunostaining of Cultured Cells

Neurospheres and dissociated cells were incubated overnight at 4°C with primary antibodies raised against the neuroepithelial cell marker nestin (rabbit, 1:2000; a gift from Dr. R. McKay, NIH, Bethesda, MD, USA); the S-phase marker BrdU (mouse 1:250, Becton Dickinson, San Jose, CA, USA, No.347580); the general neuronal marker β-III-tubulin (TuJ1 clone, mouse, 1:1,000; Covance, Berkeley, CA, USA, No. MMS-435P; and rabbit antibody, 1:300; Abcam No. ab18207); the astrocyte marker GFAP (rabbit polyclonal, 1:1,000; Dako, Glostrup, Denmark, No. Z0334; and mouse 1:1500; Millipore No. MAB360); the oligodendrocyte marker O4 (mouse monoclonal IgM, 1:8; obtained from the culture media of O4-producing hybridoma cells kindly provided by A. Rodríguez-Peña, CSIC, Madrid, Spain; and O4, 1:150; Millipore, Temecula, CA, USA, No. MAB345); and the presynaptic vesicle protein synaptophysin (rabbit polyclonal, 1:6: Invitrogen, Camarillo, CA, USA, No. 18-0130). Antibody binding to the cells was then detected using Alexa Fluor^®^ 488, 594, and Texas Red^®^ conjugated secondary antibodies (1:500, Invitrogen) and finally, nuclei were stained with Hoechst (Sigma). Controls were performed to confirm the specificity of the primary and secondary antibodies.

### Morphological Analysis of Neurons and Oligodendrocytes

A morphological analysis of TuJ1^+^ neurons was performed. From cell traces obtained using ImageJ/Fiji software (NIH), the number of primary neurites (any neurite directly extending from the cell body) and neurite branches were counted and the total neurite length and cell body perimeter were measured.

TuJ1-positive neurons were analyzed for the presence of presynaptic boutons detected with synaptophysin antibody. The number of boutons per total neurite length and the bouton perimeter were counted and measured, respectively.

The morphology of O4^+^-oligodendrocytes was analyzed as mentioned above and the number of primary processes and branches was quantified, as well as the cell network and cell body perimeter. The network perimeter was measured after tracing a closed line that touched the tip of all the processes.

### Electron Microscopy

Dissociated aOBSC neurospheres were seeded on Thermanox plastic coverslips (Thermo Fisher Scientific, 13 mm, No: 174950) covered with TRG, PLO/F, and PEI under differentiation conditions for 4, 7, and 15 DIV. Cells were then washed with 0.12 M phosphate buffer (PB) and fixed with 4% PFA + 2% glutaraldehyde for 30 min at RT. After two PB washes, the samples were post-fixed with 1% osmium tetroxide (OsO_4_) for 45 min at RT, and washed with PB. Coverslips were then dehydrated using a series of increasing alcohol concentrations where the first incubation with 70% ethanol included uranyl acetate. Finally, samples were incubated with 100% ethanol. Propylene oxide (PO) was applied for 10 min and coverslips were kept in a mix of PO + resin (Durcupan, Fluka) volume 1:1, for another 10 min before embedding in Durcupan for 24 h at RT inside a vacuum chamber. The next day, coverslips were put on top of pre-made resin columns and the resin was polymerized at 56°C for 48–72 h. Ultrathin sections (75–80 nm) were cut, counterstained with lead citrate (BDH, 29726), and studied under the electron microscope in the TEM and SEM modes.

### Real-time Quantitative Reverse Transcription Polymerase Chain Reaction

Total RNA was extracted from the OBSCs using Trizol reagent (Invitrogen) and purified with Qiagen RNeasy Mini Kit separation columns (Qiagen). The reverse transcription reaction was carried out with SuperScript III (Invitrogen) to synthesize cDNA from the mRNA.

The sequences of primer pairs for the reverse transcription polymerase chain reaction (RT-qPCR) were designed with Lasergene software and are listed in Table 2. All RT-qPCR cDNA products obtained were sequenced and corresponded to the expected fragments. A real-time qPCR analysis was performed in triplicate with the appropriate controls using Power SYBR Green (Applied Biosystems). The Ct value obtained for each target gene was normalized to the Ct value for *Gapdh* using the comparative CT method (Schmittgen and Livak, 2008).

**Table 2:** **Primers used for the gene expression profile of differentiating aOBSCs by RT-qPCR**.

Gene		Primer (5′–3′)	Size (bp)
*Ngn1*	Forward	CGATCCCCTTTTCTCCTTTCC	240
	Reverse	GTGCAGCAACCTAACAAGTG	
*Hes5*	Forward	CAGTCCCAAGGAGAAAAACCG	248
	Reverse	AGGAGTAGCCCTCGCTGTAGTC	
*Olig2*	Forward	CTGGTGTCTAGTCGCCCATC	240
	Reverse	AGGAGGTGCTGGAGGAAGAT	
*Tubb3*	Forward	CAGCGGCAACTATGTAGGGGACTC	148
	Reverse	AAAGGCGCCAGACCGAACACT	
*Gfap*	Forward	ACCAGCTTACGGCCAACAGTG	204
	Reverse	CCTCCTCCAGCGATTCAACCT	
*18S*	Forward	TCCATTATTCCTAGCTGCGGTATC	111
	Reverse	CTCGATGCTCTTAGCTGAGTGTCC	

*The PCR products have the size and sequence of the expected fragments. Ngn1, neurogenin 1; Hes5, hairy and enhancer or split 5; Olig2, oligodendrocyte lineage transcription factor 2; Tubb3, tubulin, beta 3 class III; Gfap, glial fibrillary acidic protein*.

### Cell Death Assay

The *In Situ* Cell Death Detection Kit (TMR red, Roche) was used to detect apoptosis in cultured cells by means of the terminal deoxynucleotidyl transferase (TdT)-mediated dUTP nick end labeling (TUNEL) reaction. They were incubated with BGT (3 mg/ml BSA, 100 mM Glycine, 0.25% Triton X-100), and then with a solution containing TdT and fluorescent-labeled dNTP in a 1:10 ratio. Finally, cells were washed, stained with Hoechst, and mounted.

### Cell Counts and Statistical Analysis

To determine the number of cells expressing a specific antigen or having a TUNEL^+^ label, confocal and fluorescent microscopy images of random areas in each coverslip were analyzed. For a 40× objective cells in 10 random fields and for a 20× objective cells in 5 random fields were counted per coverslip. The proportion of cells labeled by each specific marker was calculated with respect to the number of Hoechst^+^ cells and the results expressed as the average ± SEM of 3–16 cultures from 2 to 5 experiments.

Confocal images of neurospheres were acquired to analyze the number of Nestin^+^ and BrdU^+^ cells in the entire image *z*-stacks, counting the cells manually in the middle optical plane from a *z*-stack per neurosphere using ImageJ software (Wayne Rasband, NIH). We previously found that this plane was representative for the whole neurosphere (Nieto-Estévez et al., 2013). The neurosphere perimeter was also measured.

If there were two groups to compare, we used a two-tailed Student’s *t*-test with Welch’s correction when the F-test indicated significant differences between the variances of both groups. If there were more than two groups to compare, the following was done: for parametric distributions and equal variances measured by Barlett’s test, one-way ANOVA was used with Bonferroni and Tukey’s tests as *post hoc* analysis. The non-parametric Kruskal–Wallis test was used, with Dunn’s as *post hoc* test, when the variances where significantly different. For all statistical analyses, GraphPad Prism 5 software was used. The differences between the mean + SEM values were considered to be significant when *P < 0.05.

## Results

### Uncoated TRG Is a Permissive Material for the Multi-Lineage Differentiation of aOBSCs in Culture

First, the Raman spectrum of TRG showed the two well-known relative intensity bands, the D band at 1347 cm^−1^ (attributed to the presence of disorder or amorphous carbon in graphitic materials) and the G band at 1582 cm^−1^ (in-plane tangential stretching of the carbon–carbon bonds in graphene sheets) (Figure S1A in Supplementary Material). The thermal expansion of the GO to obtain TRG leaves topological defects on the graphene with the subsequent broadening of the D band. The TEM image revealed the characteristic wrinkled structure of the graphene sheet due to the thermal shock to which it has been subjected (Figure S1B in Supplementary Material). The XPS analysis indicated that TRG contained a 3.8% oxygen which is related to the presence of residual hydroxyl groups (Botas et al., 2013). Next, the physical features of the control polymers (PLO/F and PEI) and the TRG were analyzed after coverslip coating (Figure 1A). As shown, the PLO/F and PEI coverslips had a smooth surface (Figures 1B,C). The TRG/PEI-coated coverslips were examined before seeding the cells by SEM and revealed a relatively uniform distribution of the graphene sheets that almost completely covered the coverslips, and the wrinkle nanotexture resulting from the thermal reduction procedure (Figure 1D) (Schniepp et al., 2006; Ramanathan et al., 2008; Verdejo et al., 2008; Defterali et al., 2016). The TRG thickness did not exceed 1.2 nm indicating that this material consists of a few layer graphene. The distribution of the material was broadly uniform on the coverslips although some empty spots were detected (Defterali et al., 2016).

Then, the TRG/PEI-treated coverslips were also examined 4, 7, and 15 days after the initial cell plating and the TEM image shows a close and apparent direct contact between the cell processes and the TRG (Figure 1E; arrows). Cells having neuronal (arrows) or glial morphology (arrow heads) were found by SEM analysis of cultures growing on TRG and on PLO/F (Figures 1F–I). Indeed, an intact, mature-like neuron bearing branched and articulated dendrites can be visualized on the TRG-coated coverslips at 15 days (Figure 1I).

**Figure 2:**
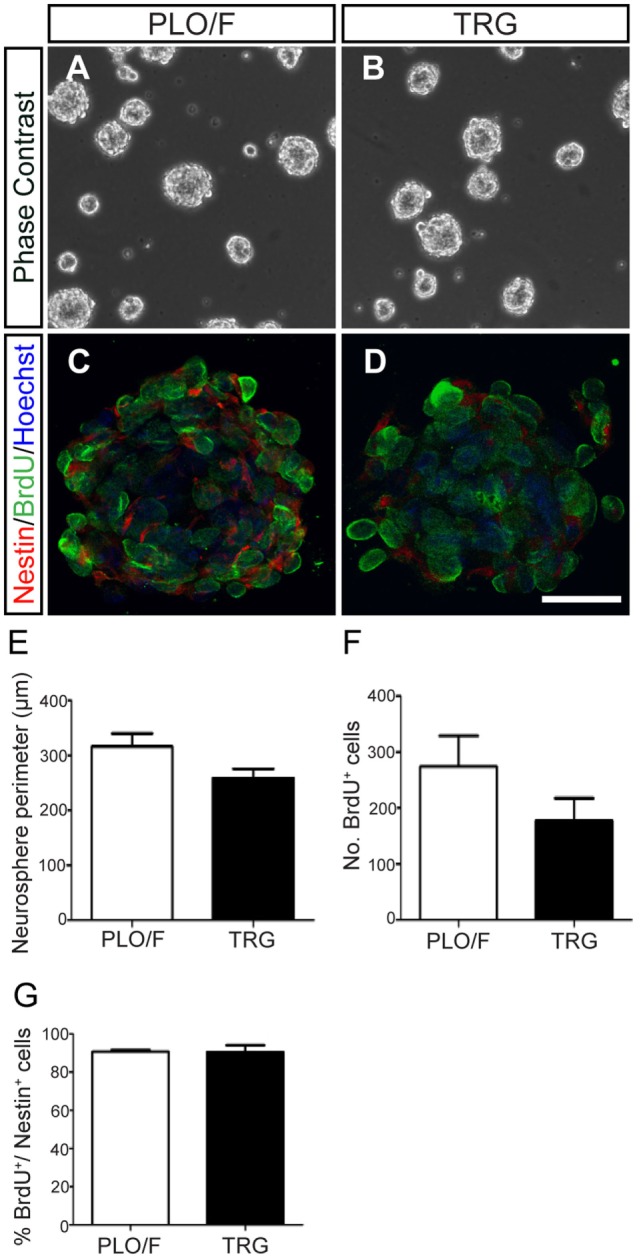
**Effect of TRG on the proliferation of aOBSCs growing as neurospheres**. The images show aOBSC neurospheres grown 3–4 days in culture on PLO/F control **(A)** and TRG coverslips **(B)**. Confocal photographs of neurosphere cross-sections (the center plane) after Nestin and BrdU immunostaining, and Hoechst nuclei counterstaining **(C,D)**. Although statistically not significant, there was a trend toward a decrease in neurosphere perimeter and in the number of BrdU^+^ cells on TRG compared to the PLO/F controls **(E,F)**. The percentages of BrdU^+^ cells over the total Nestin^+^ cells were similar **(G)**. The bars represent the mean ± SEM (*n* = 19 and 15 neurospheres for PLO/F and TRG) in **(E)**; *n* = 3 for cultures for **(F,G)**. Statistical analyses were performed using the Student’s *t*-test. Scale bar [shown in **(D)**] = 246.8 μm **(A,B)**; 30.1 μm **(C,D)**.

Next, to test the effects of graphene on neurosphere formation, cell proliferation (BrdU^+^ cells), and neuroepithelial marker expression (nestin^+^ cells), we plated dissociated aOBSCs on PLO/F- and TRG-coated coverslips, in a serum-free medium and in the presence of EGF and FGF-2. Neurospheres formed on both PLO/F and TRG cultures (Figures 2A–D). When cultured on TRG, the differences in neurosphere perimeter and the total number of BrdU^+^ cells per neurosphere were not statistically significant compared to the controls (Figures 2E,F). The large majority of cells expressed nestin and a similar proportion (~90%) of BrdU^+^ cells were seen in the neurospheres cultured in control and TRG conditions (Figure 2G). While these results confirm that dissociated aOBSCs can proliferate and form neurospheres in the presence of TRG, the diminished (yet not statistically significant) size and number of BrdU^+^ cells of these neurospheres suggest that growing neurospheres on TRG may not be advantageous compared to growing the aOBSC in our standard conditions (Vergaño-Vera et al., 2009). Accordingly, for the next experiments in which we aimed to test the effects of TRG on neuronal and glia generation from aOBSCs, as well as on the survival, differentiation, and maturation of neurons and oligodendrocytes, neurospheres were expanded in uncoated plastic dishes, then dissociated and seeded on coverslips coated with PLO/F, PEI, or TRG.

**Figure 3:**
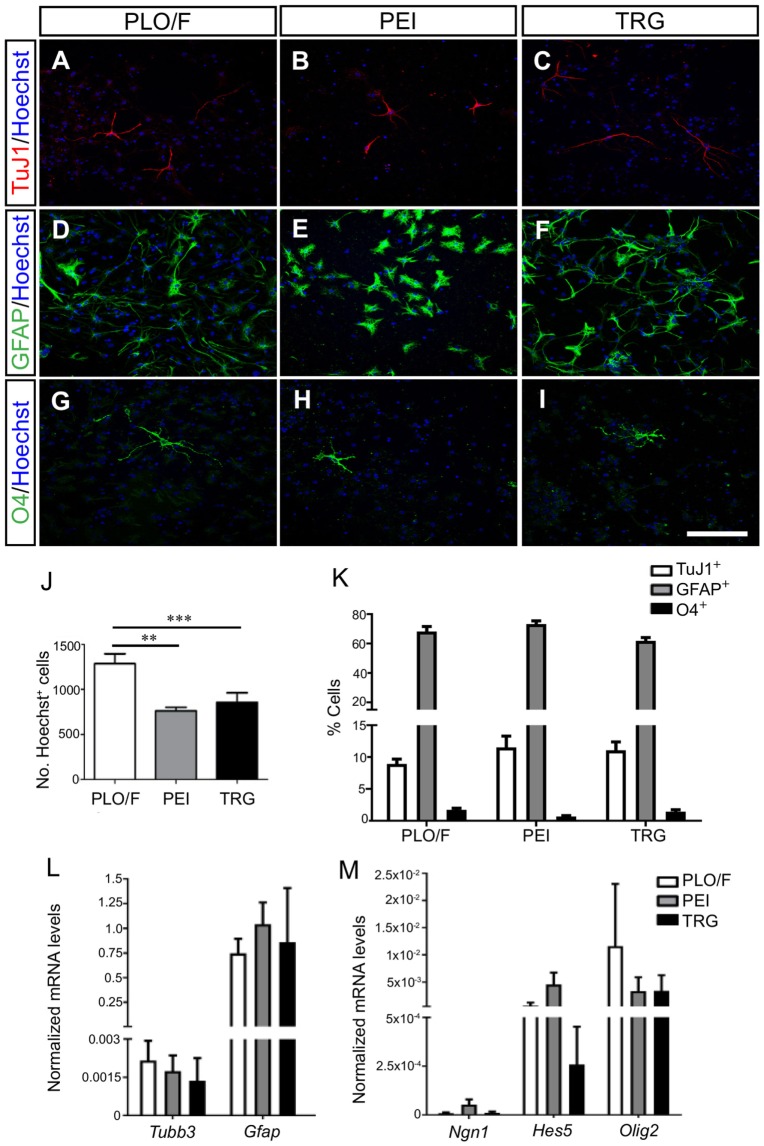
**Multi-lineage differentiation and transcription factor expression of aOBSCs grown on TRG**. The aOBSCs were grown as neurospheres, dissociated and seeded on PLO/F-, PEI-, and TRG-coated coverslips for 3–4 days in serum-free medium. They gave rise to the three major cell types of the CNS: neurons **(A–C)**, astrocytes **(D–F)**, and oligodendrocytes **(G–I)**, which were detected using antibodies against β-III tubulin (TuJ1), GFAP, and O4. Graph **(J)** shows that there was a significant decrease in the total number of cells grown on PEI- (41%) and TRG-coated (33.9%) coverslips compared to PLO/F-coated coverslips. There were no significant differences in the percentage of the three cell fates grown under all three conditions **(K)**. The expression levels of *Tubb3* and *Gfap* transcripts were similar in the three conditions **(L)**. The cell lineage-related transcripts *Ngn1, Hes5*, and *Olig2* were expressed with not significant changes between controls and TRG **(M)**. The bars represent the mean ± SEM (*n* = 4–13 cultures per condition). Statistical analyses were performed using one-way ANOVA and Tukey’s test, **P < 0.01, ***P < 0.001. Scale bar [shown in **(I)**] = 100.5 μm.

We first addressed whether TRG may influence the multi-lineage potential of aOBSCs to generate neurons (TuJ1^+^), astrocytes (GFAP^+^), and oligodendrocytes (O4^+^) (Vergaño-Vera et al., 2009). For this, dissociated aOBSCs were plated in serum-free medium for 3 days (short-term differentiation, Table 1) and immunostained (Figures 3A–I). The number of Hoechst^+^ cells growing on TRG and PEI was similar and 34–41% (*P* < 0.01) lower than on PLO/F (Figure 3J). Notably, similar percentages of each cell type (neurons, 8.9–11.5%; astrocytes, 62.0–73.3%; oligodendrocytes, 0.7–1.7%) were observed under control and TRG conditions (Figure 3K). These results were confirmed by the finding of similar expression levels of *Tubb3* and *Gfap* transcripts in the three conditions (Table 2; Figure 3L). The greater percentages of astrocytes differentiating from aOBSCs compared to those of neurons and oligodendrocytes are consistent with previous findings on the differentiation of aNSCs (Gritti et al., 2002; Sanai et al., 2004; Vergaño-Vera et al., 2009; Nieto-Estévez et al., 2013). Next, we investigated the effect of TRG on the expression of a key neurogenic gene, *Ngn1*, and of genes involved in the generation of astrocytes (*Hes5*) and oligodendrocytes *(Olig2*) (Table 2) (Vergaño-Vera et al., 2009; Díaz-Guerra et al., 2013; Imayoshi and Kageyama, 2014). Figure 3M shows that the three transcripts were expressed in cultures differentiating for 7 days (MTD, Table 1) with no significant changes being observed between conditions in controls and TRG. In summary, our results demonstrate that TRG is a permissive substrate for the multi-lineage differentiation of aOBSCs to neurons, astrocytes, and oligodendrocytes, following the appropriate neurogenic and gliogenic transcriptional programs.

### Uncoated TRG Is a Permissive Material for the Survival and Maturation of aOBSC-Derived Neurons and Oligodendrocytes

**Figure 4:**
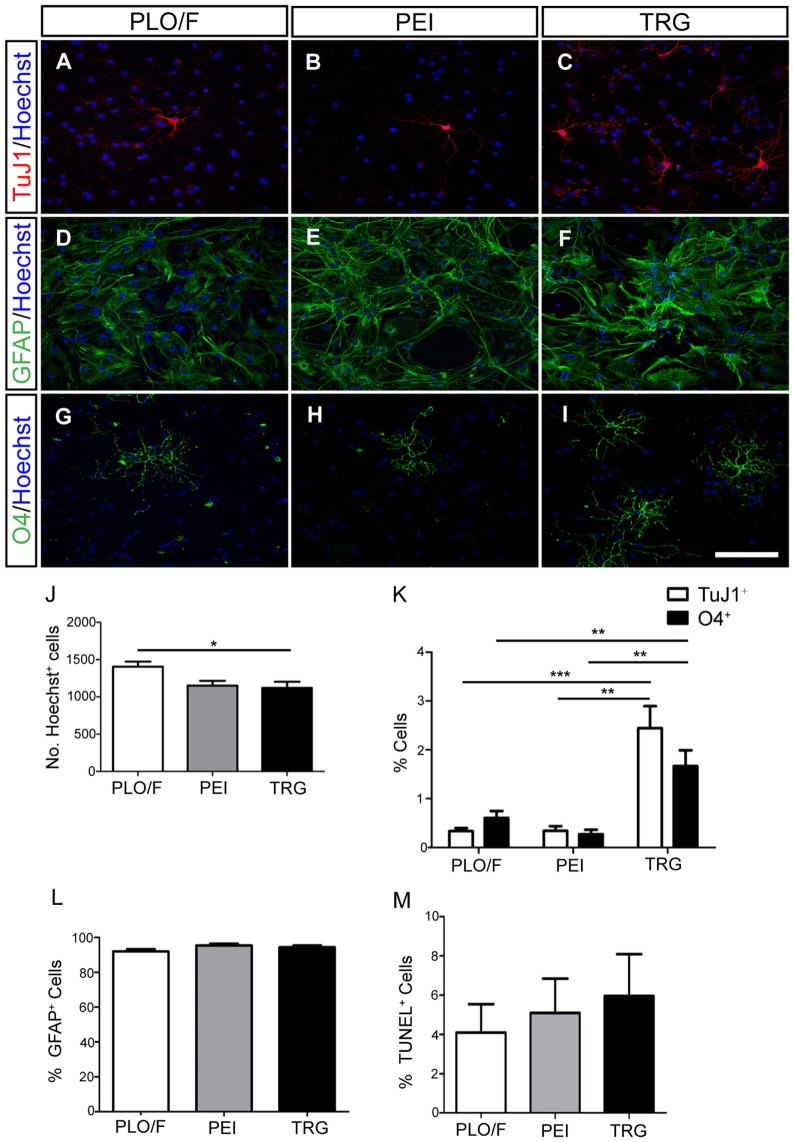
**Effect of TRG on the long-term survival of neurons, astrocytes and oligodendrocytes obtained from aOBSCs**. The aOBSC neurospheres were dissociated and seeded on PLO/F, PEI and TRG coverslips in serum-containing medium for 15–18 DIV. Neurons **(A–C)**, astrocytes **(D–F)**, and oligodendrocytes **(G–I)** were detected using antibodies against β-III tubulin (TuJ1), GFAP, and O4. A small but yet significant 15.5% decrease was found in the number of Hoechst^+^ cells grown on TRG and PEI coverslips compared to PLO/F **(J)**. There were 2- to 6-fold significant increases in the percentages of TuJ1^+^ cells and O4^+^ cells grown on TRG compared to PEI and PLO/F **(K)**. The majority of the cells in the cultures were GFAP^+^ astrocytes, and the percentages of astrocytes were similar in the three conditions **(L)**. No significant differences were observed in the percentages of TUNEL^+^ dead cells between conditions **(M)**. The graph bars represent the mean ± SEM (*n* = 5–16 cultures per condition). Statistical analyses were performed using one-way ANOVA and Kruskal–Wallis tests together with Tukey’s and Dunn’s tests. *P < 0.05, **P < 0.01, ***P < 0.001. Scale bar [shown in **(I)**] = 100.5 μm.

**Figure 5:**
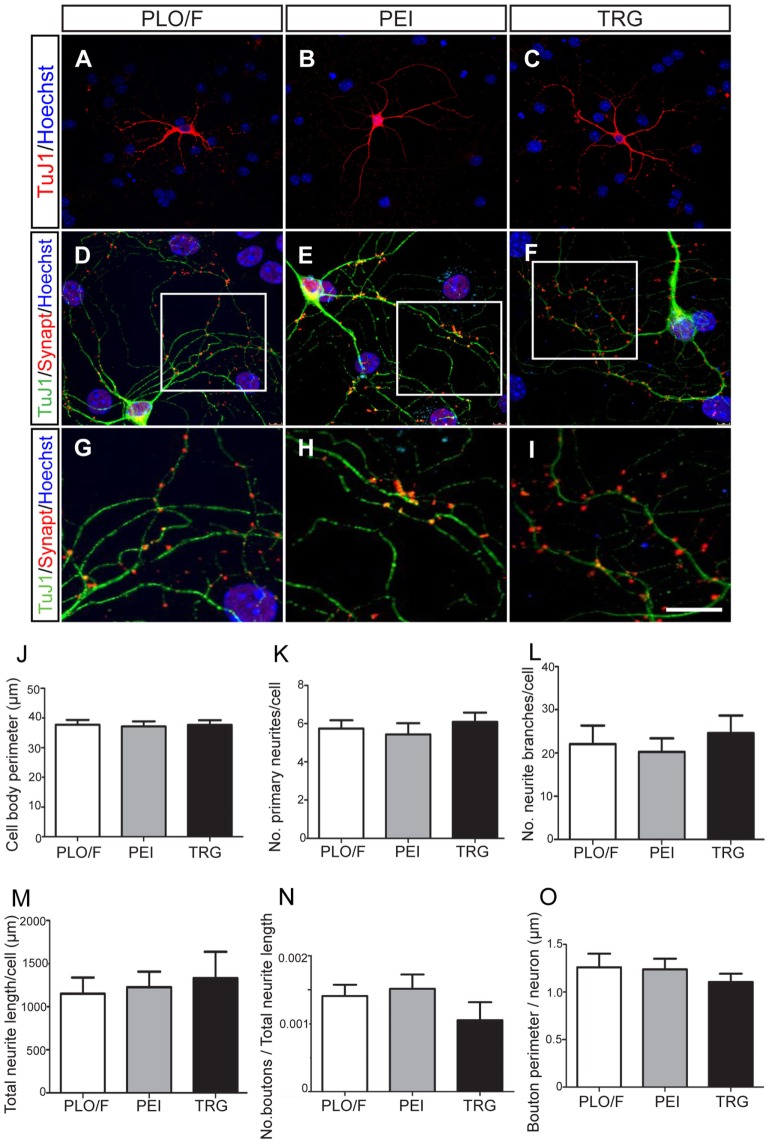
**TRG is a permissive substrate for the morphological and synaptic maturation of neurons obtained from aOBSCs**. The aOBSC neurospheres were dissociated and seeded in a serum-containing medium for 15–18 DIV. Neuronal maturation was assessed by a morphological analysis in TuJ1^+^ neurons **(A–C)**. Four different parameters were analyzed in TuJ1^+^ cells and no significant differences were found **(J–M)**. To analyze the synaptic maturation, the culture was fed 20 ng/ml of BDNF during 18 DIV. Neurons and presynaptic boutons were detected using antibodies against β-III tubulin (TuJ1) and Synaptophysin (Synapt) **(D–F)**. An area of presynaptic boutons is shown at a higher magnification in the bottom panels **(G–I)**, each corresponding to the indicated square area in the photos above. No significant differences were found in the measured parameters for synaptic maturation of cells grown on PLO/F, PEI, or TRG **(N,O)**. The bars represent the mean ± SEM (*n* = 20–31 TuJ1^+^ neurons; *n* = 15 TuJ1^+^/Synaptophysin^+^ neurons). Statistical analyses were performed using one-way ANOVA and Tukey’s test. Scale bar [shown in **(I)**] = 50.6 μm **(A–C)**, 23.4 μm **(D,E)**, 10.1 μm **(G–I)**.

**Figure 6:**
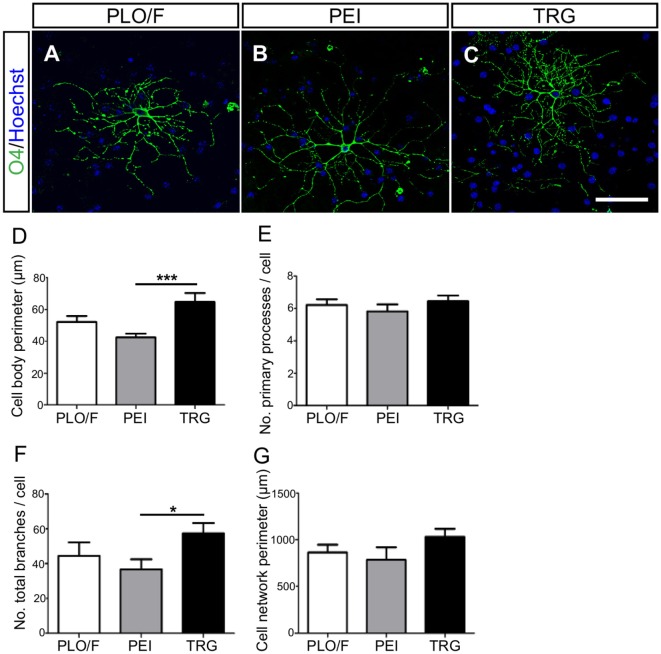
**TRG supports the morphological maturation of oligodendrocytes derived from aOBSCs**. The aOBSC neurospheres were dissociated and seeded in serum-containing medium during 15–18 DIV. Oligodendrocytes were analyzed after immunostaining with an O4 antibody **(A–C)** investigating further the cells’ morphology, using four parameters shown in the graphs **(D–G)**. There was a significant increase in the cell body perimeter **(D)** as well as in the number of total branches **(F)** of cells grown on TRG, compared to PEI. No significant differences were found in other parameters **(E,G)**. The graph bars represent the mean ± SEM (*n* = 17–32 O4^+^ oligodendrocytes). Statistical analyses were using one-way ANOVA and Tukey’s test. *P < 0.05, ***P < 0.001. Scale bar [shown in **(C)**] = 50.6 μm.

To investigate the effect of TRG on the long-term survival, morphological differentiation, and synaptic maturation of cells generated from aOBSCs, the cells were plated and maintained for 15–18 days (LTD, Table 1) in culture media containing 2% FBS to enhance cell survival and morphological differentiation (Figures 4, 5A–C,J–M and 6) or 2% FBS plus BDNF to enhance synaptic maturation (Figures 5D–I,N,O) (Vicario-Abejón et al., 1998). The total number of Hoechst^+^ cells counted on TRG coverslips was similar to PEI and 15.5% lower (*P* < 0.05) compared to PLO/F (Figure 4J). In contrast, marked and significant increases (2- to 6-fold; *P* < 0.05 to *P* < 0.001) in both the numbers (not shown) and percentages of TuJ1^+^ neurons (Figures 4A–C,K) and O4^+^ oligodendrocytes (Figures 4G–I,K) growing on TRG were observed when compared to PLO/F or PEI conditions. The majority of the cells in the cultures were GFAP^+^ astrocytes, as mentioned above, and the percentages of astrocytes were similar in the three conditions (Figures 4D–F,L) although the numbers of GFAP^+^ astrocytes were lower in PEI and TRG than in PLO/F (PLO/F: 163.2 ± 9.2, PEI: 128.3 ± 12.0, TRG: 119.1 ± 32.3 GFAP^+^ cells; *n* = 9). These data suggest that the decrease in total Hoechst^+^ cell number in PEI and TRG could be attributable to a reduction in the astrocyte population. As seen in Figure 4M the percentages of TUNEL^+^ dead cells were not statistically different between TRG, PEI, and PLO/F. These findings show that in a serum-containing medium, graphene favors the long-term survival of neurons and oligodendrocytes and it is a permissive substrate for astrocytes derived from aOBSCs.

A series of morphological changes are associated with the cellular mechanisms underlying the differentiation and maturation of neurons and oligodendrocytes (Dotti et al., 1988; Vicario-Abejón et al., 1995, 2000; Jan and Jan, 2010; Lafrenaye and Fuss, 2010; Vinet et al., 2010; Vergaño-Vera et al., 2015). Accordingly, we performed a morphological analysis of TuJ1^+^ neurons cultured on PLO/F, PEI, and TRG and found that the values for the cell body perimeter, number of primary neurites, number of branches, and total neurite length were similar in the three conditions suggesting that neurons can morphologically differentiate equally well in all conditions (Figures 5A–C,J–M). Remarkably, the neurons developed to express synaptophysin (a marker of presynaptic maturation) accumulated in boutons, suggesting that they have the potential to form synapses (Figures 5D–I). The number and size of boutons were similar in the three conditions (Figures 5N,O), indicating that TRG is a permissive substrate for the morphological and synaptic maturation of aOBSC-derived neurons.

The morphology of O4^+^-oligodendrocytes was also analyzed (Figures 6A–G). When compared to PEI control, oligodendrocytes grown on the TRG substrate had larger cell body perimeter (52%, *P* < 0.001) and more branches (56%, *P* < 0.05) (Figures 6D,F). However, the changes observed comparing TRG values versus PLO/F control were not statistically significant. No effects of TRG were observed in other parameters tested (Figures 6E,G). Oligodendrocytes generated on TRG resembled type I and II oligodendrocytes, with approximately six primary processes that branched repeatedly and acquired a complex morphology (Lafrenaye and Fuss, 2010; Vinet et al., 2010). These results suggest that TRG but not PEI favors the morphological maturation of oligodendrocytes.

### Pristine PVDF Membranes as well as Membranes Loaded with MWCNTs Are Permissive Substrates for Neuronal Survival but Reveal Conflicting Results for Morphological Differentiation of Neurons

**Figure 7:**
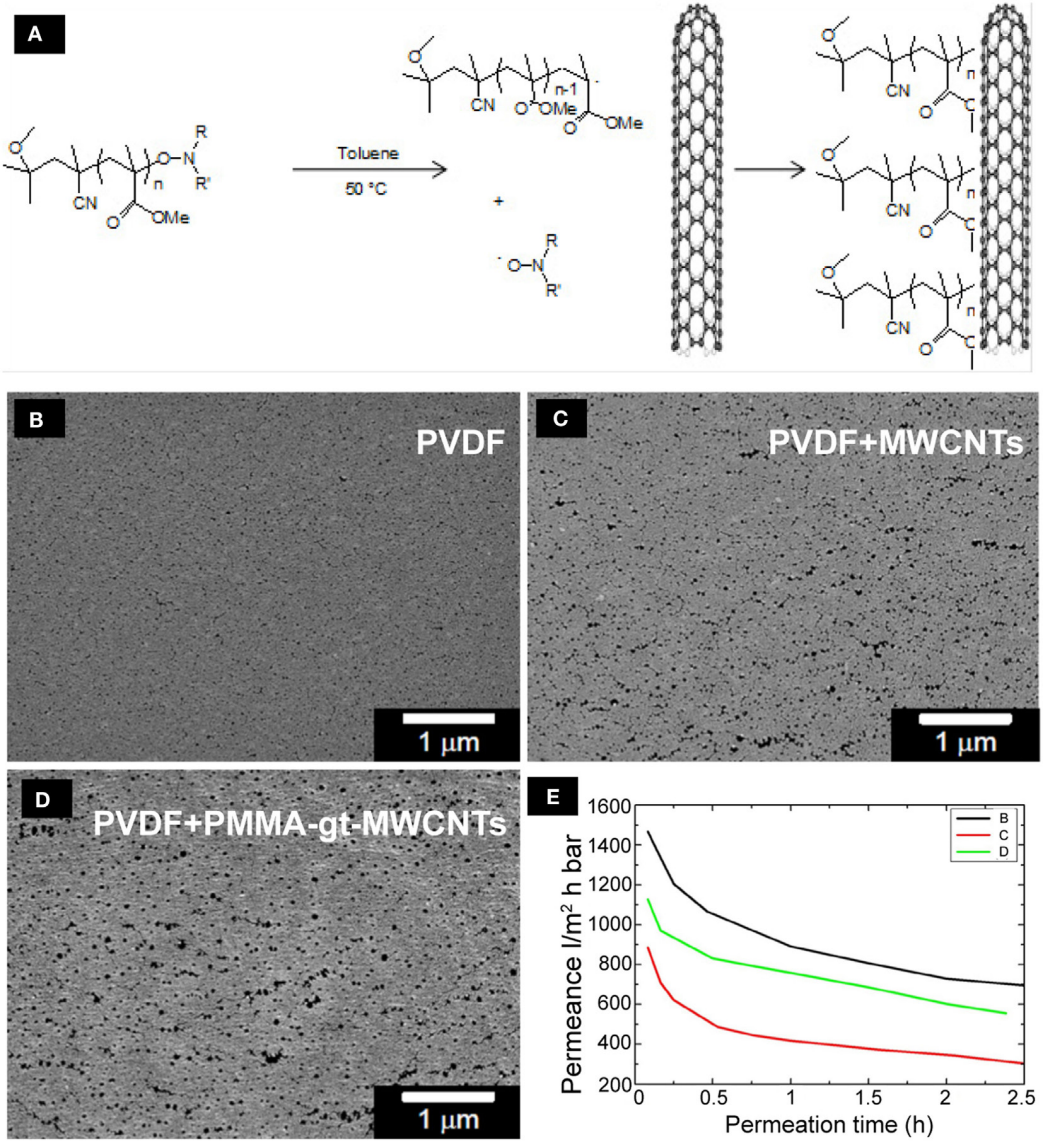
**The functionalization and distribution of MWCNTs on PVDF membranes**. Schematic diagram of the grafting of alkoxyamine end-capped PMMA chains onto MWCNTs **(A)**. SEM microphotographs of the top view of pristine PVDF membranes **(B)**, PVDF membranes + MWCNTs **(C)**, and PVDF membranes + PMMA-gt-MWCNTS **(D)** are shown. The PVDF layer has been loaded with 2 wt% of MWCNTs with and without grafted polymers. The graph **(E)** shows water permeation measurements of the PVDF membranes.

The characterization of functionalized MWCNTs by TGA analysis showed that PMMA levels grafted onto MWCNTs were 7.74 wt% (Figure S2 in Supplementary Material; Table 3). Then, the morphology of the membranes (Table 3) was examined by optical microscopy of the particles in the membranes, prior to cell seeding (Figure S3 in Supplementary Material). The optical micrographs show the dispersion state observed when the MWCNTs were modified by the “grafting to” technique with 7.7 wt% of PMMA (Figure 7A). Scanning electron micrographs in Figures 7C,D show that the morphology of membranes loaded with MWCNTs is different from the one of a pristine PVDF membrane (“standard HZG,” Figure 7B). The membranes prepared with MWCNTs (Figures 7C,D) showed bigger pores and, in some cases, the interconnection of pores.

**Table 3:** **Details of the membranes prepared**.

Sample	Polymer	Polymer amount (g)	Nanoparticle	MWCNT loading with respect to the polymer (wt%)
10/019	PVDF	80	MWCNT-purified	2.0
10/022	PVDF	20	PMMA-gt-MWCNTs (7.7 wt% of grafted polymer by “grafting to”)	2.0

The highest porosity was observed on the top layer of the PVDF membranes containing PMMA-gt-MWCNTs, which is particularly interesting since this membrane presented the lowest bubble point value among the nanocomposite membranes (Table 4). This indicates that the apparent pore size along the membrane thickness does not correspond to the size observed on the top layers.

**Table 4:** **Bubble point values for PVDF membranes**.

Sample	Bubble point (μm)
PVDF	0.18 ± 0.02
PVDF_MWCNTs-purified	0.29 ± 0.03
PVDF_PMMA-gt-MWCNTs	0.19 ± 0.01

Despite the possibility of the occurrence of fouling in the membrane during water permeation, the flux decay observed in Figure 7E is mainly explained by the exchange of isopropanol and water inside the membrane. Once the alcohol used for conditioning is washed out to a certain extent, the water flux stabilizes and no further fouling is observed. The water permeance of the pristine PVDF membranes was higher compared to loaded membranes. Membranes containing purified MWCNTs showed the lowest water permeance, which might be due the agglomerates shown in optical micrographs (Figure S3 in Supplementary Material). The agglomerates might have partially hindered the pore formation, resulting in a reduction in water permeance of the membranes. Membranes containing PMMA-gt-MWCNTs showed higher water permeation compared to the membranes incorporated with purified MWCNTs, which might be due to lesser WCNT agglomerates, as can be seen in Figure S3 in Supplementary Material.

**Figure 8:**
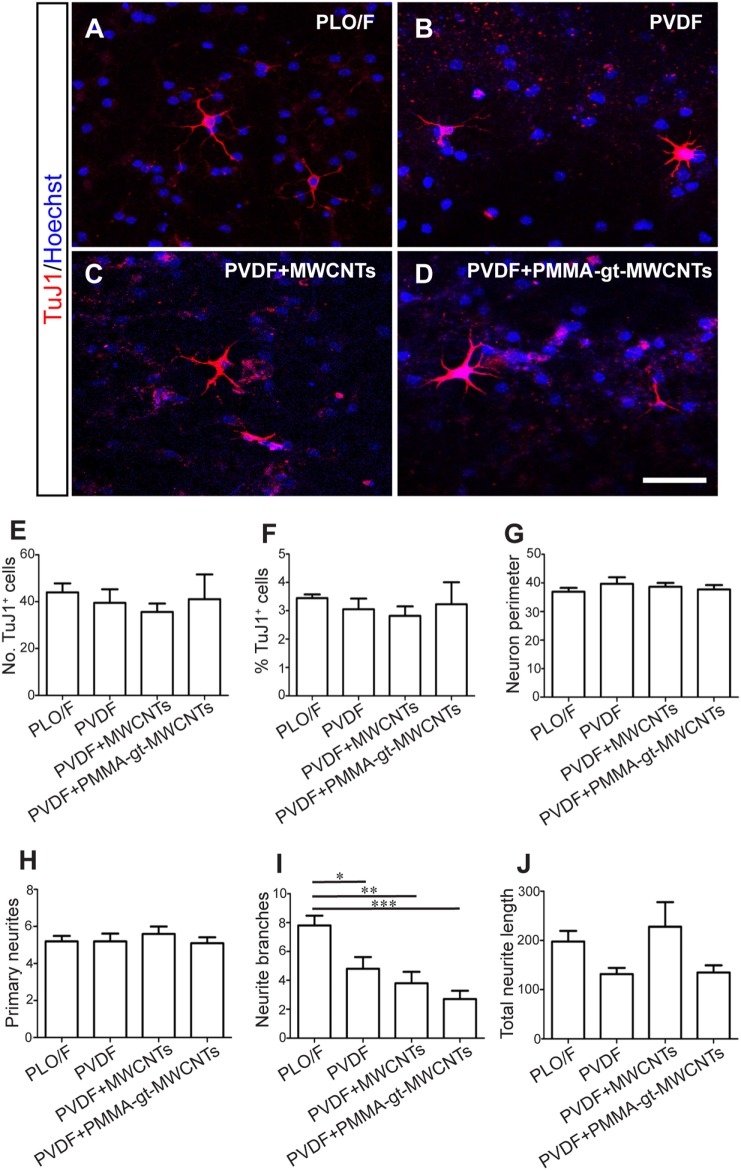
**PVDF membranes are permissive substrates for neuronal survival but inhibit neurite branching**. The aOBSC neurospheres were dissociated and seeded on PLO/F, and PVDF, PVDF + MWCNTs and PVDF + PMMA-gt-MWCNTs membranes in serum-containing medium for 7 days and formed neurons, which were detected by antibodies against β-III tubulin (TuJ1) **(A–D)**. Graphs show no significant differences in the numbers **(E)** and percentages **(F)** of TuJ1^+^ neurons grown on all conditions. There were no significant differences in the neuron perimeter, the primary neurites, and the total neurite length **(G,H,J)**. Compared to the PLO/F, neurons had significant decreases in neurite branches when grown on pristine and loaded PVDF membranes **(I)**. The graph bars represent the mean ± SEM (*n* = 5–6 cultures per condition; *n* = 10 TuJ1^+^ neurons). Statistical analyses were performed using one-way ANOVA and Tukey’s test as *post hoc* analysis. *P < 0.05, **P < 0.01, ***P < 0.001. Scale bar [shown in **(D)**] = 31.5 μm.

**Figure 9:**
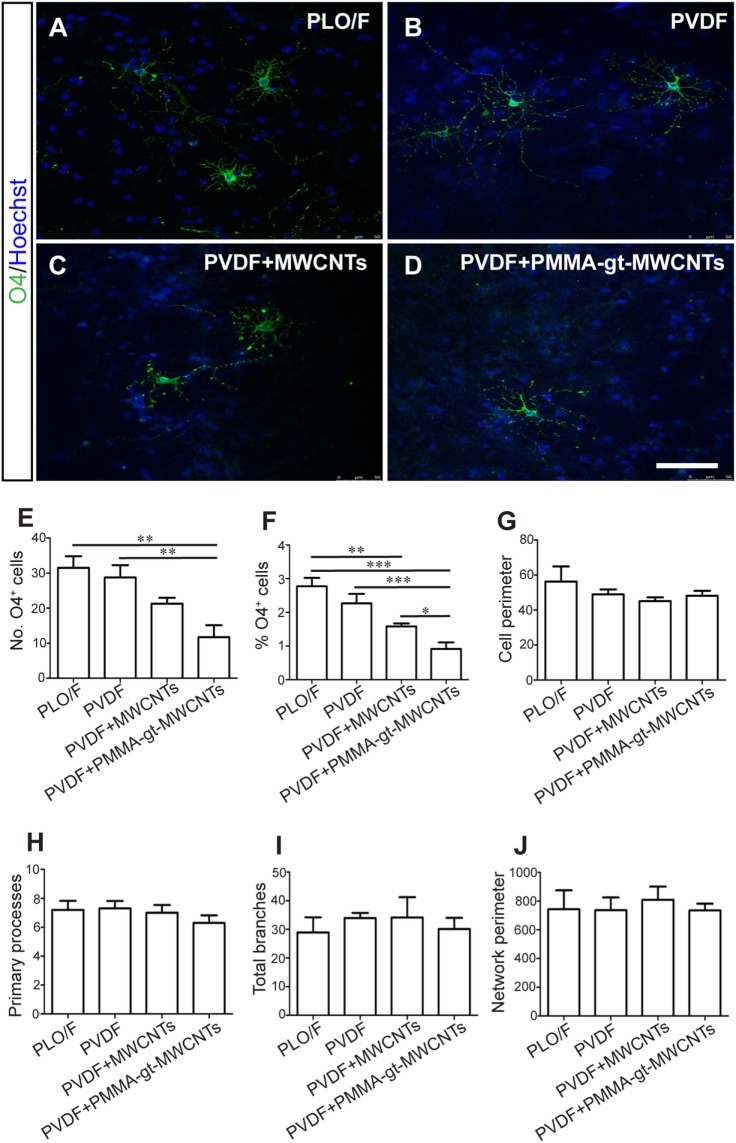
**PVDF membranes do not support oligodendrocyte survival but are permissive for their morphological differentiation**. The aOBSC neurospheres were dissociated and seeded on PLO/F, and PVDF, PVDF + MWCNTs, and PVDF + PMMA-gt-MWCNTs membranes for 7 days in a serum-containing and formed oligodendrocytes, which were detected by antibodies against O4 **(A–D)**. There were significant decreases in the number of O4^+^ oligodendrocytes when grown on PVDF + PMMA-gt-MWCNTs compared to PVDF and PLO/F **(E)**. For the percentage of oligodendrocytes **(F)**, there were significant decreases when cells were grown on PVDF + MWCNTs and PVDF + PMMA-gt-MWCNTs, compared to PLO/F and PVDF **(F)**. There were no significant differences in morphological parameters between conditions **(G–J)**. The graph bars represent the mean ± SEM (*n* = 5–6 cultures per condition; *n* = 10 O4^+^ oligodendrocytes). Statistical analyses were performed using one-way ANOVA and Kruskal–Wallis tests and Tukey’s and Dunn’s tests as *post hoc* analysis, respectively. *P < 0.05, **P < 0.01, ***P < 0.001. Scale bar [shown in **(D)**] = 48.1 μm.

Once the membranes were characterized, aOBSCs growing as neurospheres were dissociated and seeded on PLO/F and PVDF membranes (Figures 8 and 9). The cells were then grown for 7 days (MTD, Table 1) and neurons were detected using antibodies against β-III tubulin (TuJ1) (Figures 8A–D). Graphs show no significant differences in the numbers (Figure 8E) and percentages (Figure 8F) of TuJ1^+^ neurons grown on all conditions. For the morphological parameter analysis, the neuron perimeter, the primary neuritis, and the total neurite length did not differ significantly between conditions (Figures 8G,H,J). There was a significant 38.5, 51.3, and 65.4 decrease in neurite branches when cells were grown on PVDF, PVDF + MWCNTs, and PVDF + PMMA-gt-MWCNTs compared to PLO/F, suggesting that the membranes had an inhibitory effect on neurite branching. Differentiation of GFAP^+^-astrocytes was observed in all conditions (data not shown).

### PVDF Membranes Loaded with MWCNTs Are Not Ideal Substrates for Oligodendrocyte Survival but Permissive for Their Morphological Differentiation

Cells were grown using the same protocol as described above and oligodendrocytes were detected by antibodies against O4 (Figures 9A–D). There were significant decreases in both the number and percentages of O4^+^ oligodendrocytes when grown on PVDF + PMMA-gt-MWCNTs compared to PVDF and PLO/F (Figures 9E,F). However, the reductions observed in PVDF + MWCNTs were significant only when expressed in percentages and were compared to PLO/F (Figures 9E,F). In addition, there was a significant 42.2% oligodendrocyte decrease when cells were grown on PVDF + PMMA-gt-MWCNTs, compared to PVDF + MWCNTs (Figure 9F). No significant changes between conditions were observed in the morphology of oligodendrocytes.

## Discussion

Graphene, GBNs, and CNTs are being investigated as potential suitable materials for the growth of NSCs, neurons, and glial cells and for their ability to modulate the function of neuronal circuits *in vitro* (Li et al., 2011, 2013; Park et al., 2011; Lorenzoni et al., 2013; Solanki et al., 2013; Tang et al., 2013; Shah et al., 2014; Gupta et al., 2015; Vicentini et al., 2015; Weaver and Cui, 2015; Guo et al., 2016c; Marchesan et al., 2016). However, in these previous studies cells were plated on those materials previously coated with extracellular matrix proteins, which are known to promote cell adhesion, differentiation, and neurite outgrowth (Vicario et al., 1993; Calof et al., 1994; Specht et al., 2004; Otaegi et al., 2006; Nishimune et al., 2008). To the best of our knowledge, no studies reporting the biocompatibility of uncoated graphene with aNSCs and aNSC-derived neurons and glia have yet been published (Bendali et al., 2013; Sahni et al., 2013; Lee et al., 2015; Fabbro et al., 2016; Veliev et al., 2016). Here, we have studied the biocompatibility of uncoated TRG with aOBSCs and aOBSC-derived with neurons, astrocytes, and oligodendrocytes. Our findings indicate that TRG is a permissive material that allows for the generation of neurons and glial cells from aOBSCs. They also show that TRG supports the morphological differentiation of oligodendrocytes and the synaptic maturation of neurons. In contrast, both pristine PVDF membranes and membranes loaded with MWCNTs reported conflicting results regarding the survival and/or differentiation of aOBSC-derived neurons and oligodendrocytes.

Our results show that aOBSCs can proliferate and form neurospheres on TRG although our BrdU analysis reveals a lower yet not statistically significant number of BrdU^+^ cells for neurospheres growing on TRG. In addition, TRG allows for the multi-lineage differentiation of aOBSCs into neurons, astrocytes, and oligodendrocytes and the expression of neuronal and glial related genes, indicating that this material does not alter a cardinal feature of NSCs, namely, their capacity to give rise to neurons and glia (Vicario-Abejón et al., 2003; Vergaño-Vera et al., 2009). Furthermore, not only does TRG permit but it may also promote the long-term survival of neurons and oligodendrocytes derived from aOBSCs when they are grown in a medium containing 2% FBS. This condition could improve solubility and biocompatibility of graphene due to protein (such as albumin) adsorption to this material, favoring cell survival and growth (Shi et al., 2014; Marchesan and Prato, 2015; Oliveira et al., 2015). The reasons for the increase in neuronal survival could also be due to the fact that the material’s electrical conductivity value is similar to what was previously reported (Schniepp et al., 2006; Zhao et al., 2012; Defterali et al., 2016) and the close contact between the material and the cells that was observed in our TEM analysis. Electrical coupling has also been proposed as a mechanism to explain the enhanced neuronal differentiation of NSCs in laminin-coated graphene (Park et al., 2011; Tang et al., 2013). In fact, electrical currents stimulate NSC differentiation into neurons (Chang et al., 2011a; Akhavan et al., 2016) and electrical activity is known to affect neuronal differentiation and survival in both the developing and adult nervous system (Spitzer, 2006, 2012). It could also be plausible that the topological patterns of the TRG with a characteristic wrinkle nanotexture might favor cell survival and oligodendrocyte morphological maturation. In support of this, we do not observe the same augment in neurons and oligodendrocytes on PEI-treated coverslips, which have a smooth surface compared to TRG. Positive actions of the presence of ripples and wrinkles have also been claimed to explain the promoting effects of laminin or poly-l-lysine-coated graphene or graphene nanogrids on neurite sprouting and oligodendrocyte differentiation (Li et al., 2011; Solanki et al., 2013; Shah et al., 2014). However, the specific instructive physical cues for these actions are yet to be discovered.

In contrast to the aforementioned beneficial effects of TRG on aOBSCs, PEI alone and uncoated TRG appear not to be sufficient to maintain the total number of cells in the cultures; an effect probably due to the reduced attachment of the cells to PEI. However, the previous coating of the coverslips with PEI was necessary to attach the TRG to the glass coverslip, as mentioned above. The decrease in cell number was less significant when the cells were differentiating in a serum-containing medium, a condition that favors cell adhesion and growth (Vicario-Abejón et al., 1998; Vergaño-Vera et al., 2009). Indeed, under this culture condition aOBSC-derived neurons developed synaptophysin-positive boutons when grown on TRG. Since synaptophysin is a key presynaptic protein for neurotransmitter release (Vicario-Abejón et al., 2002), our results suggest that neurons could potentially form functional synapses on TRG.

In contrast to TRG, our results show that PVDF membranes (pristine and loaded with MWCNTs) did not support neurite branching. Furthermore, membranes loaded with purified MWCNTs and PMMA-gt-MWCNTs were not suitable for oligodendrocyte survival. These deleterious effects could be due to the presence of impurities (such as metallic nanoparticles and amorphous carbon) in the membranes and/or to properties related to water permeance and suboptimal hydrophilicity. In support of the later idea, the poorest neurite branching and oligodendrocyte survival was observed in loaded PVDF membranes that were less permeable to water than the pristine ones. In contrast, high hydrophilicity can accelerate differentiation of NSCs into neurons (Akhavan et al., 2014). In addition to the reduced permeance of loaded PVDF membranes, the functionalization of MWCNTs with 7.7% PMMA (that leaves free ketone, ether, and cyano groups) did not favor cell growth as demonstrated by the significantly lower percentage of O4^+^ cells and the trend of reduced neurite branching in PVDF + PMMA-gt-MWCNTs compared to PVDF + MWCNTs membranes. These results also indicate that aOBSC-derived oligodendrocytes are more sensitive to reactive oxygen and cyano groups than aOBSC-neurons.

In line with our observation of diminished cell attachment to both PEI and TRG, it was very recently reported that primary neurons can survive and differentiate on peptide-free graphene but their cell number was decreased, probably due to the reduced cell adherence to the uncoated material (Bendali et al., 2013; Sahni et al., 2013). Our data show that TRG does not significantly change the number of TUNEL^+^ dead cells, suggesting that the reduced cell attachment due mainly to PEI may be the primary cause for the partial loss of cells seeded on TRG and PEI compared to PLO/F.

In conclusion, our findings indicate that uncoated TRG does not have a deleterious effect on adult OB cells *in vitro*. Indeed, TRG may be a permissive substrate for the multi-lineage differentiation of aOBSCs into neurons, astrocytes, and oligodendrocytes as well as for neuronal synaptic maturation. In contrast, PVDF membranes (both pristine and loaded with PMMA-gt-MWCNTs) were not permissive for neurite branching and oligodendrocyte survival. These conclusions might have important implications for the study of the interaction of TRG- and MWCNT-loaded PVDF membranes with functional neuronal networks.

## Author Contributions

ÇD: design, collection, and assembly of data, data analysis and interpretation, and manuscript writing. RV: conception and design, graphene preparation and characterization, data collection, manuscript writing, and financial support. SM: membrane preparation and characterization, collection and assembly of data, and data analysis and interpretation. AB-d-F: design, collection and assembly of data, and data analysis and interpretation. HM-G: collection of data. ED-G: collection of data and data analysis and interpretation. DF: membrane preparation and characterization. KB: membrane characterization. CA: membrane characterization. RM-M: data collection. DV: membrane modification and characterization. MA: design, membrane modification and characterization. J-MT and CD: membrane modification and characterization. CJ and VA: design and financial support. ML-M: conception and design, graphene preparation and characterization, manuscript writing, and financial support. CV-A: conception and design, data analysis and interpretation, financial support, manuscript writing, and final approval of manuscript.

## Conflict of Interest Statement

The authors declare that the research was conducted in the absence of any commercial or financial relationships that could be construed as a potential conflict of interest.
