# Supplementary material for: In Vitro Evaluation of Biocompatibility of Uncoated Thermally Reduced Graphene and Carbon Nanotube-Loaded PVDF Membranes with Adult Neural Stem Cell-Derived Neurons and Glia
Source: Front Bioeng Biotechnol. 2016 Dec 6;4:94. doi: 10.3389/fbioe.2016.00094 (PMC5138223; doi:10.3389/fbioe.2016.00094)
Supplement: Supplementary file 1 [file Data_Sheet_1.docx]

**Fig. S1. Characterization of TRG by Raman spectrometry and TEM**

A) The Raman spectrum of TRG shows the D band at 1347 cm^-1^ (attributed to the presence of disorder or amorphous carbon in graphitic materials) and the G band at 1582 cm^-1^ (in-plane tangential stretching of the carbon–carbon bonds in graphene sheets). B) The TEM image displays the characteristic wrinkled structure of the graphene sheet due to the thermal shock during its preparation. TEM, transmission electron microscopy.

**Fig. S2. Characterization of functionalized MWCNTs by TGA**

The graph shows that grafted PMMA to MWCNTs represented a 7.74% of the functionalized MWCNT weight. Sample references for analysis were neat MWCNTs and pure PMMA without nanotubes. TGA, thermogravimetric analysis.

**Fig. S3. Optical microphotographs of MWCNTs dispersed in PVDF matrix** (2 wt % MWCNTs loading with respect to PVDF). MWCNTs-purified (A) and PMMA-gt-MWCNTs (5.8 wt % of grafted polymer by “grafting to“) (B).
