# Supplementary figures and images for: In Vitro Evaluation of Biocompatibility of Uncoated Thermally Reduced Graphene and Carbon Nanotube-Loaded PVDF Membranes with Adult Neural Stem Cell-Derived Neurons and Glia
Source: Front Bioeng Biotechnol. 2016 Dec 6;4:94. doi: 10.3389/fbioe.2016.00094 (PMC5138223; doi:10.3389/fbioe.2016.00094)

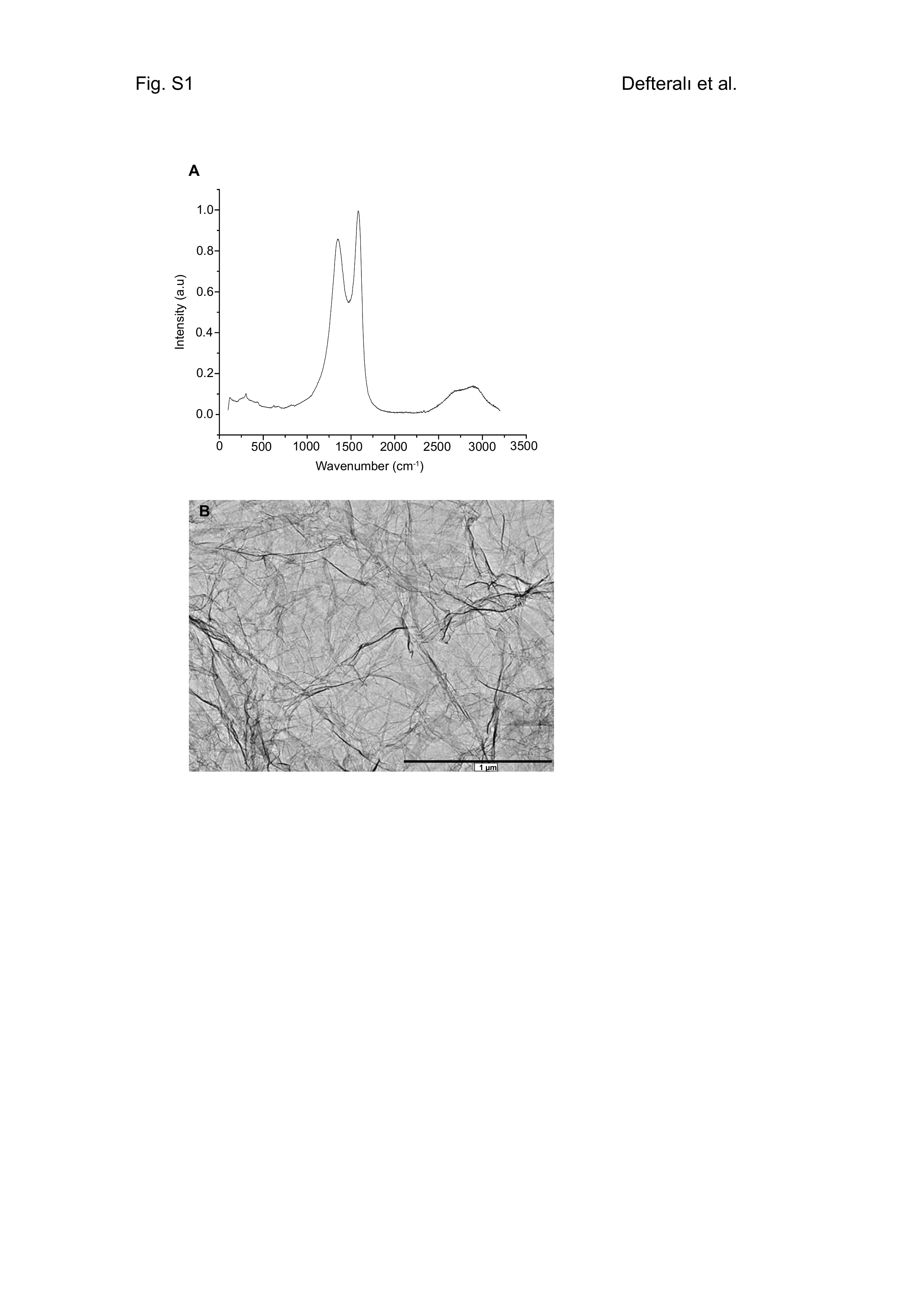

Supplement: Supplementary file 2 [file Image_1.TIF]

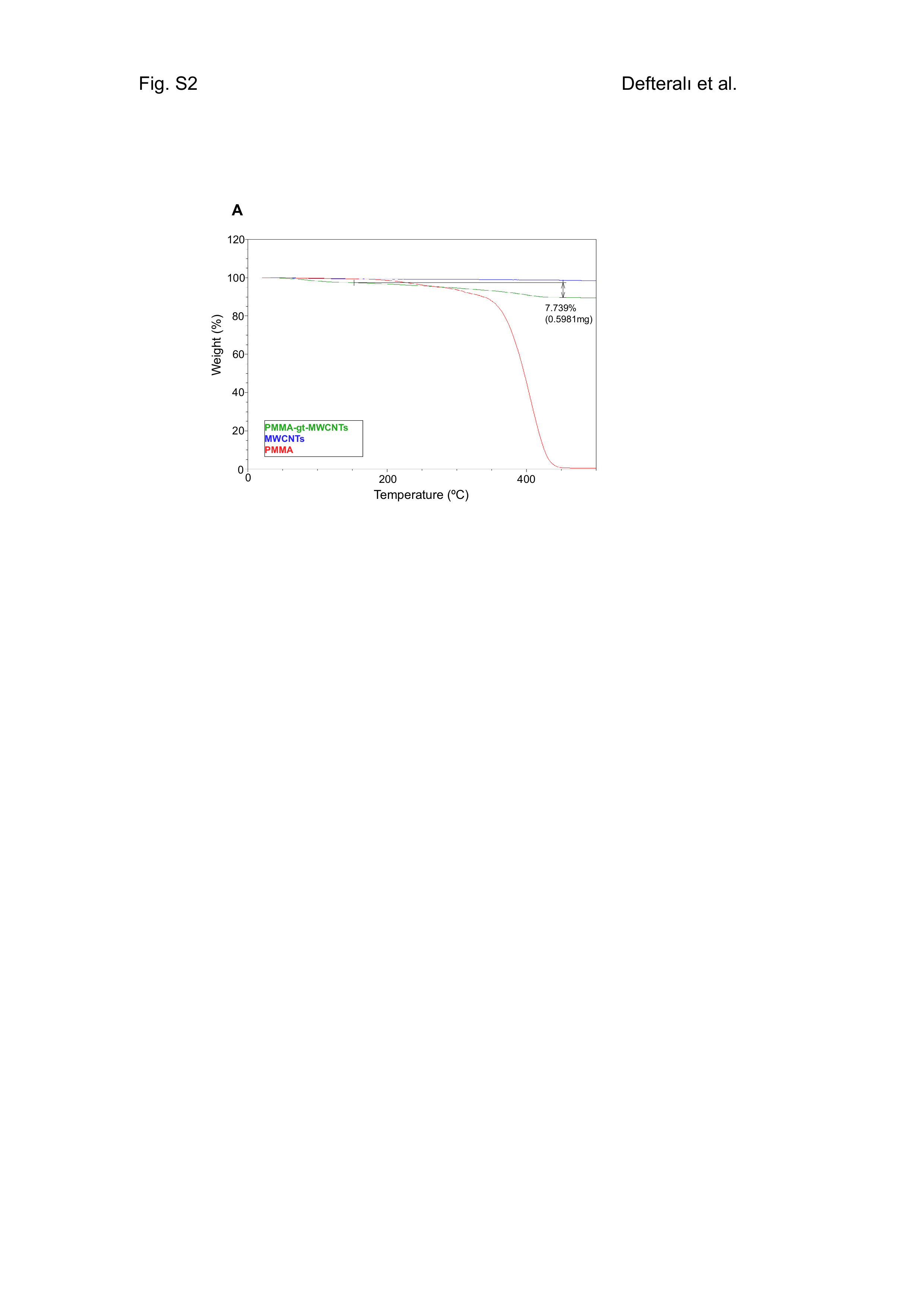

Supplement: Supplementary file 3 [file Image_2.TIF]

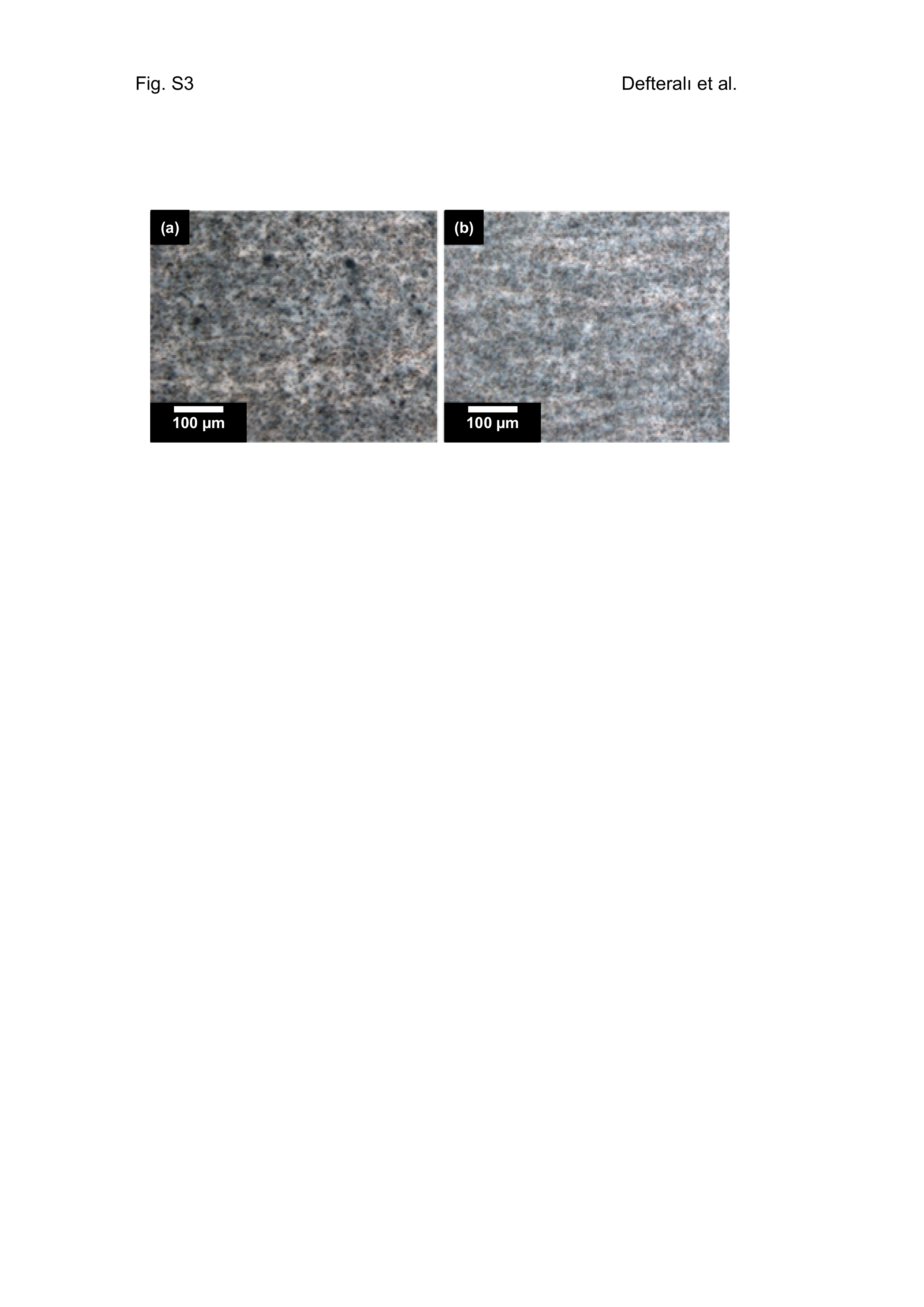

Supplement: Supplementary file 4 [file Image_3.TIF]
